# A Case of Warm Autoimmune Hemolytic Anemia and a Pulmonary Embolus in a Patient Treated with Triple Therapy

**DOI:** 10.1155/2019/2564682

**Published:** 2019-08-26

**Authors:** Gurchetan Randhawa, Chia-Yu Chiu, Thanunthorn Suban Na Ayutthaya

**Affiliations:** ^1^Maimonides Medical Center, Department of Medicine, Brooklyn, NY, USA; ^2^Lincoln Medical Center, Department of Medicine, The Bronx, NY, USA

## Abstract

Autoimmune hemolytic anemia (AIHA) can be caused by a variety of etiologies. AIHA is associated with the development of coagulopathy, leading to potentially fatal pulmonary emboli. Here, we present a case of a 66-year-old female with a past medical history of non-Hodgkin's lymphoma and gastritis treated with triple therapy that developed warm AIHA. The patient later succumbed to a suspected pulmonary embolus.

## 1. Introduction

Approximately one-half of warm AIHAs are idiopathic, while the nonidiopathic etiologies include lymphoproliferative disorders, autoimmune disease, and medication-induced AIHA. Medication-induced AIHA is a syndrome where a medication acts as a hapten on the surface of red blood cells (RBC) causing a sequence of hemolysis. IgG antibodies attach to the medication-RBC complex at body temperature, and the complex is transported to the spleen where it is consumed by macrophages. This, in turn, produces spherocytes, which are then destroyed by the spleen, thus causing hemolysis. This is contrast to cold AIHA, where IgM is the implicated immunoglobulin, and the complex forms at below core body temperature.

The association between non-Hodgkin's lymphoma and AIHA has been well established in the literature. It is possible that non-Hodgkin's lymphoma acts as a paraneoplastic syndrome producing antibodies against the surface of RBCs although the exact pathophysiology remains controversial.

## 2. Case Presentation

A 66-year-old female with a past medical history of hypertension, stage IIIA diffuse large B-cell lymphoma (DLBCL), and *H. pylori* gastritis presented to the emergency department complaining of epigastric pain for one week. The patient was recently treated for *H. pylori* gastritis (confirmed via esophagogastroduodenoscopy) with triple therapy but returned to the emergency department due to recurrent pain. She denied the presence of blood in her stool. The pain is worse with eating, is associated with a metallic taste, and prevented her from eating for the past ten days. She could only complete ten out of the fourteen days of her triple therapy regimen consisting of: amoxicillin 500 mg twice a day, clarithromycin 500 mg twice a day, and lansoprazole 30 mg twice a day.

The patient was treated for DLBCL three years ago with six cycles of R-CHOP (rituximab, cyclophosphamide, doxorubicin, vincristine, and prednisone) therapy. She attained a complete response and was on surveillance; however, a follow-up chest computed tomography (CT) scan one month prior to her admission indicated the presence of nine bilateral pulmonary nodules. The etiology of the nodules was unknown; however, they were concerning for recurrent lymphoma, primary lung malignancy, and metastatic disease.

Her physical exam was remarkable for dry oral mucosa and epigastric tenderness. Her lab studies were remarkable for leukocytosis (WBC: 18.5 K/*μ*L), macrocytic anemia (Hgb: 5.1 g/dL, Hct: 13.8%, MCV: 122.7 fL), and LDH: 551 U/L. A fecal occult blood test was negative. A direct antiglobulin test (DAT) was IgG positive and C-3 negative. A peripheral smear indicated the presence of spherocytes and hypersegmented neutrophils. She received 3 units of packed RBCs and prednisone 70 mg (1 mg/kg) on the first day of admission.

On her second day of admission, the patient complained of dyspnea with oxygen saturation between 70 and 80% on 2 L nasal cannula. Her vital signs were temperature: 98.7°F, blood pressure: 140/90 mmHg, heart rate: 120 beats/min, and respiratory rate: 25 breaths/min. On physical exam, she had jugular venous distention with bibasilar crackles; however, she denied calf pain and the presence of varicose veins. She was switched to BiPAP with improvement in oxygen saturation to 98%. Due to the patient's rapid deterioration, a bedside point-of-care ultrasound was performed and indicated the presence of a dilated right ventricle (positive D sign with septal flattening) on the parasternal short axis view. A chest X-ray indicated cephalization of the pulmonary vasculature as well as right atrial enlargement. An initial electrocardiogram (EKG) indicated the presence of an S1 Q3 T3 pattern. The patient was intubated due to hypoxia. The clinical suspicion for a pulmonary embolus was high based upon these acute findings. Possible etiologies for the suspected pulmonary embolus include infection, recent blood transfusion, glucocorticoid use, immobility, AIHA, and malignancy.

Approximately ten minutes postintubation, the patient became hypotensive and bradycardic. The patient received a repeat EKG that demonstrated pulseless electrical activity, and she received cardiopulmonary resuscitation. She was given tissue plasminogen activator (tPA), and her oxygen saturation only improved after completing tPA therapy. She continued to receive resuscitation for approximately 35 minutes before regaining a pulse and was transferred to the medicine intensive care unit. Upon arrival to the intensive care unit, the patient went into cardiac arrest once again, and despite medical intervention, the patient expired.

## 3. Discussion

Warm AIHA is more common than cold AIHA, and it accounts for approximately 75% of all AIHAs in adults. The signs and symptoms of autoimmune hemolytic anemia are nonspecific, and the patient may initially present with weakness, dyspnea, jaundice, and tachycardia.

Laboratory findings of patients with warm AIHA include positive direct antiglobulin testing (Coombs test), spherocytes on peripheral blood smears, reticulocytosis, elevated lactate dehydrogenase (LDH), elevated indirect bilirubin, and decreased haptoglobin. At the behest of the patient's family, an autopsy was not performed; therefore, the true etiology of AIHA and the pulmonary nodules remains unknown. However, we suspect that the AIHA was medication-induced or malignancy-induced.

The most common medications to cause AIHA are antibiotics. The American Red Cross collected data in Southern California from 2000 to 2009 and reported 83 episodes of medication-related warm AIHA. The most commonly implicated agents were cefotetan (36 cases), ceftriaxone (17 cases), and piperacillin (14 cases) [1]. Another review article published in 2015 from a single center in Germany analyzed data from 1996 to 2015. They reported 73 cases of medication-related AIHA. Their most implicated agents were diclofenac (23 cases), followed by piperacillin (13 cases), ceftriaxone (12 cases), and oxaliplatin (10 cases) [2].

The first documented case of amoxicillin-induced autoimmune hemolytic anemia was reported in 1985 [3]. To the best of our knowledge, our case is the first documented case in which a patient developed warm AIHA while being treated with triple therapy (amoxicillin, clarithromycin, and lansaprazole). Although unreported in the literature, antibodies to clarithromycin or lansoprazole should also be considered as potential offending agents implicated in warm AIHA.

The association between venous thromboembolism (VTE) and AIHA has been well established in the literature. One meta-analysis from 2015 indicated that patients with AIHA had a 2.6-fold higher risk of developing VTE when compared with non-AIHA patients. The risk was more pronounced in the first year after diagnosis [4].

The mechanism of coagulation activation in hemolytic anemia is unclear and likely multifactorial. Normally, phosphatidylserine is found in the inner monolayer of the cell membrane. Destruction of the RBC membrane by autoantibodies leads to alteration of RBC membrane organization. This results in a loss of membrane phospholipid asymmetry and increased expression of anionic phospholipids, especially phosphatidylserine. This process causes an increased risk for coagulation and the development of VTE [5, 6].

AIHA are associated with lymphoproliferative disorders, the most common being chronic lymphocytic leukemia (CLL) [7]. In one retrospective study from 2010 involving 960 patients with CLL, 7% had autoimmune cytopenia, of which 19 were detected at diagnosis, 3 before diagnosis, and 48 during the course of the disease. 49 patients had autoimmune hemolytic anemia, 20 patients had immune thrombocytopenic purpura, and 1 patient had both conditions [8]. Both Hodgkin's and non-Hodgkin's lymphoma have also been associated with AIHA [9].

Treatment approaches for warm AIHA include reduction in autoantibody production and effectiveness. Glucocorticoids, cytotoxic agents, and rituximab are used to reduce autoantibody production. To reduce autoantibody effectiveness, splenectomy or intravenous immune globulin are utilized. Intravenous immune globulin acts by decreasing the interaction between splenic macrophages and antibody-coated RBCs [10].

Initial management includes stabilization of the patient (e.g., blood transfusion), administration of glucocorticoids as first-line agents, and searching for secondary causes (e.g., offending medications and contributing disorders). The recommended initial doses of glucocorticoids are 1 to 1.5 mg/kg per day. For patients with rapidly evolving, severe hemolysis, intravenous methylprednisolone 250–1000 mg/day for 1–3 days or 100–200 mg/day for 10–14 days is recommended. Once remission is achieved, the glucocorticoid dose is gradually tapered to find the optimal, lowest dose that maintains remission. Patients with warm AIHA should be treated for a minimum of 3-4 months with prednisone ≤10 mg/day. Bisphosphonates, vitamin D, calcium, and folic acid should also be given with long-term glucocorticoid therapy. Once hemoglobin, haptoglobin, LDH, and the absolute reticulocyte count are normalized, glucocorticoids can be discontinued, even with a positive Coombs test [11].

Second-line therapies include splenectomy and rituximab. Since there are no randomized trials comparing these therapies, the choice of splenectomy vs. rituximab relies upon the healthcare provider. Rituximab, a monoclonal antibody against CD20, has been successfully used in treatment-resistant AIHA as a single agent or in conjunction with glucocorticoids [10].

Third-line agents include immunosuppressive and cytotoxic agents, such as cyclophosphamide and azathioprine. The initial dose of cyclophosphamide is 100 mg/day orally or 500–700 mg intravenously every 3-4 weeks. The initial dose of azathioprine is 100–150 mg/day orally. For cases refractory to prednisone and splenectomy, high doses of intravenous gamma globulin (1000 mg/kg per day for five days) may be utilized [11].

## 4. Conclusion

In summary, this is a case of a 66-year-old woman with warm AIHA after 10 days of treatment with triple therapy for *H. pylori* gastritis. She also had bilateral pulmonary nodules of unclear etiology, which were concerning for the presence of malignancy, possibly due to a recurrent DLBCL treated 3 years prior. The patient eventually succumbed on the second day of admission with clinical findings consistent with a pulmonary embolus. Although the etiology of this patient's AIHA remains unclear, we hope that our paper can highlight the development of AIHA, as well as its potentially devastating complication of VTE formation.
